# Detrimental effects of long-term elevated serum uric acid on cognitive function in rats

**DOI:** 10.1038/s41598-021-86279-y

**Published:** 2021-03-24

**Authors:** Tian Tian, Xi-run Liu, Ting-ting Li, Zhi-chao Nie, Shuang-jing Li, Yan Tang, Cong-wei Gu, Wang-dong Xu, Hong Jia

**Affiliations:** 1 School of Public Health, Southwest Medical University, Luzhou, 646000 China; 2 Clinical Drug Trial Institution, The Affiliated Hospital of Southwest Medical University, Luzhou, 646000 China; 3 Laboratory Animal Centre, Southwest Medical University, Luzhou, 646000 China; 4 Collaborative Innovation Centre for Prevention of Cardiovascular Research of Sichuan Province, Key Laboratory for Medical Electrophysiology of Ministry of Education, Luzhou, 646000 China

**Keywords:** Cognitive neuroscience, Learning and memory

## Abstract

Uric acid is a powerful antioxidant. However, its elevated levels in association with cardiovascular diseases predispose individuals to cognitive impairment. Uric acid’s effects on cognition may be related to its concentration and exposure period. We aimed to explore the effects of long-term elevated serum uric acid on cognitive function and hippocampus. Rats were randomly divided into four groups: NC, M1, M2 and M3 groups. Hyperuricemia was established in rats at week 6 and maintained until week 48 in groups M1, M2 and M3. The rats’ spatial learning and memory abilities were assessed by the Morris Water Maze test at weeks 0, 6, 16, 32, and 48. After week 48, we observed pathological changes in right hippocampal CA1 and CA3 regions, and measured levels of oxidative stress, inflammatory cytokines, and β-amyloid peptide of left hippocampus. Starting from week 6, the serum uric acid level of M3 group > M2 group, the serum uric acid level of M2 group > M1 group, and the serum uric acid level of M1 group > NC group. The rats in M3 and M2 groups had longer escape latencies, longer mean distances to the platform, more extensive pathological damage, stronger inflammation response, higher oxidative stress and β-amyloid peptide levels than those in NC group. No significant differences were observed between M1 and NC groups. In addition, we also found that oxidative stress significantly correlated with tumour necrosis factor-α and β-amyloid peptide. Long-term elevated serum uric acid was significantly associated with cognitive impairment risk. Oxidative stress, tumour necrosis factor-α and β-amyloid peptide may mediate the pathogenesis of the cognitive impairment induced by uric acid. The detrimental effect of elevated serum uric acid on cognitive function was probably expressed when the serum uric acid concentration reached a certain level.

## Introduction

Uric acid is an end-product of purine metabolism generated during the enzymatic degradation of xanthine and excreted from the kidneys and bowels. Elevated serum uric acid (SUA) is associated with several cardiovascular diseases and often contributes to cognitive impairment morbidity^1^. Cognitive impairment is a chronic neurodegenerative condition characterized by poor learning and memory^2^. It is estimated that about 22% of the elderly > 70 years of age have some degree of cognitive impairment in the United States^3^. With the aging of our society, cognitive impairment is becoming a serious public health problem. More studies are required to identify the risk factors of cognitive impairment.

Recently, several studies investigated the association between uric acid and cognitive impairment with inconsistent results. In theory, uric acid is an endogenous antioxidant, accounting for about two-thirds of the plasma antioxidant power in humans, which is thought to exert a neuroprotective effect^4^. Findings from several studies support this hypothesis. Several cross-sectional or case–control studies have suggested that an increased SUA level is related to a decreased risk of cognitive dysfunction^5–7^. In a prospective study, a higher baseline SUA level was associated with subsequently enhanced cognitive performances, even in the special cognitive domain^8,9^. Similar results have also been demonstrated in the early stage of cognitive dysfunction. A large cross-sectional study on 2102 Chinese elderly individuals has shown a linear decrease of mild cognitive impairment prevalence with the increase of SUA levels^10^.

However, the antioxidant capacity of the uric acid remains questionable in several studies. The elevated SUA level has been linked to several chronic disease states, including obesity, metabolic syndrome, diabetes, hypertension, non-alcoholic fatty liver disease and cardiovascular events^11–14^. SUA decreased in gout patients treated with pegloticase (a recombinant uricase), but the plasma markers of oxidative stress remained unchanged^15^. These conflicting results indicate that higher SUA levels are related to poor cognitive performances. The Rotterdam Scan Study found that elevated SUA was related to white matter atrophy and worse cognition^16^. In a prospective cohort study, higher baseline SUA was associated with faster cognitive decline over time in a visual memory/visual construction ability test^17^.

Overall, the association between uric acid and cognitive impairment is complex. Findings from previous studies were inconsistent. Recently, in Huang et al. study, generalized additive models revealed a U-shaped carve relationship between SUA and cognitive function. Still, the further analysis found no significant positive association between increased mild cognitive impairment risk and elevated SUA among subjects whose SUA was above the cut-off point (388.63 µmol/L)^18^. The uric acid impact on cognitive function may go through a chronic development process. It may be impossible to observe the uric acid’s detrimental effect on cognition while in low concentration or during short-term exposure. In this study, we aimed to explore the effects of long-term elevated SUA on the cognitive function and hippocampus.

## Methods

### Ethics statement

The Ethics Committee for Animal Research of the Southwest Medical University approved the whole experiment. Animals were handled in strict accordance with good animal practice. During the experiment, all animal-related procedures were performed following relevant guidelines and regulations of the Ethics Committee for Animal Research of the Southwest Medical University. All procedures involving live animals were also performed observing the Animal Research: Reporting of In Vivo Experiments (ARRIVE) guidelines.

### Animals

Thirty-two male Sprague-Dawley rats (213 ± 14.21 g weight, 8 months old) were provided by the Laboratory Animal centre of the Southwest Medical University. The rats were housed two per cage in clean cages under controlled temperature (23 ± 2 °C), relative humidity (55 ± 5%), and light–dark cycle (light, 08:00–20:00 h; dark, 20:00–08:00 h). Access to food and water was unrestricted. The animals were pacified before and after each experiment.

### Hyperuricemia rats

To establish hyperuricemia models, rats were fed yeast and/or potassium oxonate diets^19,20^. Yeast contains abundant protein and a nucleotide, which can increase uric acid production. Potassium oxonate, a uricase inhibitor, can inhibit uric acid excretion. The combined application of yeast and potassium oxazinate improve the increasing of SUA levels. Rats were randomly divided into four groups of eight rats: NC group, M1 group, M2 group and M3 group. NC group was the control group with a normal diet. M1 group was the control group with yeast diet, and M1 group rats were fed a yeast diet containing 1% yeast per day. M2 group was the control group with a potassium oxonate diet, and M2 group rats were fed a potassium oxonate diet containing 0.025% potassium oxonate per day. M3 group was the positive group with yeast and potassium oxonate diet, and M3 group rats were fed a diet containing 1% of yeast and 0.025% potassium oxonate per day. We persistently provided yeast and potassium oxonate diets to rats. The rats were anesthetized by intraperitoneal injection of sodium pentobarbital 30 mg/kg, and blood (1 ml) was sampled from rat heart in vivo at weeks 0, 6, 16, 32 and 48. The blood was centrifuged for collect serum, the SUA level were measured by automatic biochemical analyzer (Beckman, USA).

### Morris water maze test

The rats’ spatial learning and memory abilities were evaluated by the Morris water maze (MWM) test, as described previously^21^, before each blood draw. In the orientation-navigation test, each rat was allowed to swim and search for a platform within 120 s in 5 consecutive days. The escape latency, swimming speed and mean distance to the platform were recorded. If the rat failed to find the platform, the escape latency was recorded as 120 s. In the spatial probe test, the platform was removed on the 6th day. Each rat was allowed to swim within 120 s, and the frequency with which the rat passed the original platform quadrant was recorded. All trials were conducted between 10:00 a.m. and 13:00. The behavioural differences of the rats in each MWM test among the groups were evaluated. At week 48, the escape latencies and mean distances to the platform among groups showed a significant difference, and the rats stopped receiving food. The 48th-week indexes served as final data.

### Histopathology

After week 48, the rats were anesthetized with 40 mg/kg pentobarbital sodium and then humanely killed to collect the hippocampus. The right hippocampus was fixed in 4% paraformaldehyde solution and cut into sections. The sections stained with haematoxylin–eosin (HE) were used to observe the CA1 and CA3 regions’ morphology and structure. The sections stained with terminal deoxynucleotidyl transferase-mediated nick end labelling (TUNEL) were used to monitor the CA1 and CA3 regions’ apoptotic cells. Apoptotic cells are pyramidal cells with prominent yellowish-brown granules or plaques in the nuclei. Three non-overlapping fields of CA1 and CA3 regions in each section were randomly selected to monitor the apoptotic cells and calculate the apoptosis indexes (apoptosis index = the number of apoptotic cells/the number of total cells × 100%) under a light microscope (400×), respectively.

### An enzyme-linked immunosorbent assay (ELISA)

Left hippocampus tissue and iced physiological saline were mixed in a volume ratio of 1:9 and then added to a tissue homogenizer for homogenization. The homogenate was centrifuged at 4000 rpm for 15 min to obtain a supernatant used for the ELISA test.

The superoxide dismutase (SOD) levels were measured using an ELISA kit (Jiancheng, Nanjing, China) at an absorbance of 450 nm, and the detection limit was 0.5 U/ml. The malondialdehyde (MDA) levels were measured using an ELISA kit (Jiancheng, Nanjing, China) at an absorbance of 532 nm. The glutathione peroxidase (GSH-Px) levels were measured using an ELISA kit (Jiancheng, Nanjing, China) at an absorbance of 412 nm. The β-amyloid peptide (Aβ) levels were measured using an ELISA kit (Duqiao, Shanghai, China) at an absorbance of 450 nm, and the detection limit was 1.0 ng/ml. The interleukin-1β (IL-1β) levels were measured using an ELISA kit (Duqiao, Shanghai, China) at an absorbance of 450 nm, and the detection limit was 0.1 pg/ml. The tumour necrosis factor-α (TNF-α) levels were measured using an ELISA kit (Duqiao, Shanghai, China) at an absorbance of 450 nm, and the detection limit was 1.0 pg/ml. All experimental operations complied with the manufacturer’s protocol. All samples were measured in duplicates, and plates were read automatically using a full-wavelength microplate reader (Molecular Devices, USA). Concentration was calculated based on a standard linear curve.

### Statistical analysis

SPSS 24.0 was used for all statistical analyses. Data with normal distribution were presented as mean value ± standard deviation and analysed with Analysis of variance. Variables without normal distribution were presented as median (25th percentile, 75th percentile) and analysed with the Kruskal–Wallis H test. Repeated measures analysis of variance was used to evaluate the difference of repeated measurement data among the groups. The associations between SUA, oxidative stress, inflammation cytokines and Aβ were analysed using Pearson’s correlation analysis. P-value < 0.05 was established as statistically significant. GraphPad 6.0 was used to draw graphs.

## Results

### SUA levels

A rat with an SUA value > 110 µmol/L was considered a hyperuricemia rat^22^. The SUA levels in M3 and M2 groups were 148.24 ± 21.99 µmol/L and 142.56 ± 21.21 µmol/L, respectively, at week 6, and the SUA level in the M1 group was 136.93 ± 19.22 µmol/L at week 16, indicating that we successfully induced hyperuricemia rat models in the M3, M2 and M1 groups.

**Figure 1:**
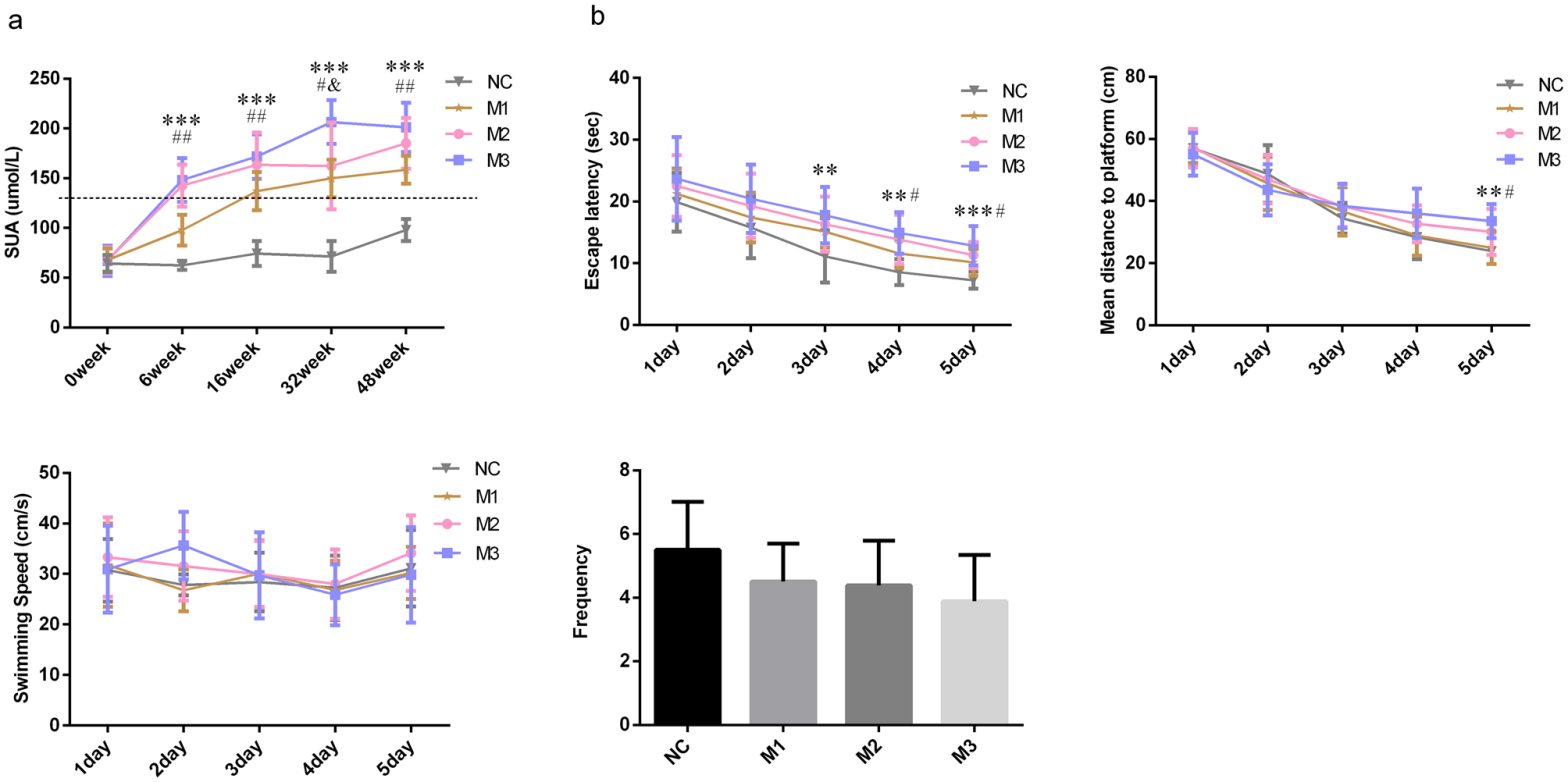
SUA levels and MWM indicators of rats. Data presented as mean ± standard deviation, n = 8 for each group. *P < 0.05 compared to the NC group, ^#^P < 0.05 compared to the M1 group, and ^&^P < 0.05 compared to the M2 group.

From week 6, the SUA levels in M3, M2 and M1 groups were persistently increasing. At week 6, the SUA levels of rats in the M3 group were higher than those in the M1 group (*P* < 0.001) and NC group (*P* < 0.001), the SUA levels of rats in the M2 group were higher than those in the M1 group (*P* < 0.001) and NC group (*P* < 0.001), the SUA levels of rats in the M1 group were higher than those in the NC group (*P* < 0.001). At week 16, the SUA levels of rats in the M3 group were higher than those in the M1 group (*P* = 0.004) and NC group (*P* < 0.001), the SUA levels of rats in the M2 group were higher than those in the M1 group (*P* = 0.025) and NC group (*P* < 0.001), the rats’ SUA levels of the M1 group were higher than those in the NC group (*P* < 0.001). At week 32, the rats SUA levels in the M3 group were higher than those in the M2 group (*P* = 0.003), M1 group (*P* < 0.001), and NC group (*P* < 0.001), and the SUA levels of rats in the M2 group (*P* < 0.001) and M1 group (*P* < 0.001) were higher than those in the NC group. At week 48, the SUA concentrations of rats in the M3 group were higher than those in the M1 group (*P* < 0.001) and NC group (*P* < 0.001), and the SUA levels of rats in the M2 group were higher than those in the M1 group (*P* = 0.013) and NC group (*P* < 0.001), the SUA concentrations of rats in the M1 group were higher than those in NC group (*P* < 0.001). In general, starting from week 6, the SUA level of the M3 group > M2 group, the SUA level of the M2 group > M1 group, and the SUA level of the M1 group > NC group (Fig. 1a).

### Morris water maze

The escape latency and mean distance to the platform can reflect the learning ability of the animal. At week 48, the reduction in escape latency and mean distance to the platform were observed in all rats in 5 days. But the performance comparison among the groups was difficult. The rats in the 4th group had comparable swimming speed (F = 0.708, *P* = 0.555). The escape latencies (F = 3.093, *P* = 0.043) and mean distances to the platform (F = 3.196, *P* = 0.039) in the four groups were different. The escape latencies in the M3 (*P* = 0.007) and M2 (*P* = 0.035) groups were more extended than that in the NC group. The rats in the M3 (*P* = 0.023) and M2 (*P* = 0.041) groups had worse performance in the mean distance to the platform than those in the NC group. The mean distance in the M1 (*P* = 0.035) group was also shorter than that of the M3 group. The frequency of the rat passing the original platform quadrant indicated the degree of memory consolidation which had taken place after learning. The rats had comparable frequencies among groups (F = 1.896, *P* = 0.153), but the frequencies in M3, M2 and M1 groups were all lower than the NC group. In general, the rats in M3 and M2 groups had longer escape latencies and mean distances to the platform than those in the NC and M1 groups, indicating that the rats’ spatial learning ability in the M3 and M2 groups were worse than those in the NC and M1 groups (Fig. 1b).

### Histopathology analysis

**Figure 2:**
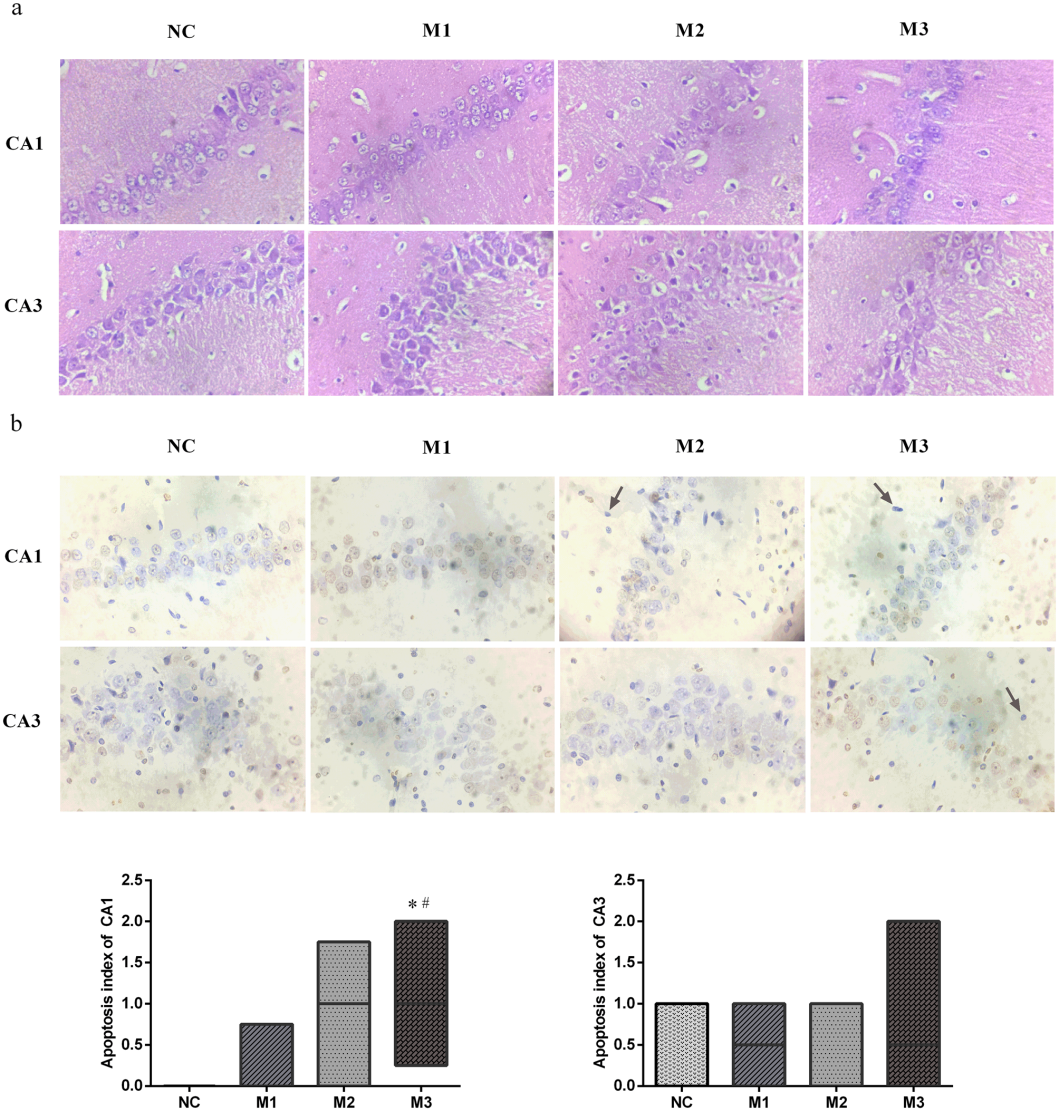
The pathological changes of hippocampal CA1 and CA3 regions of rats. (**a**) Haematoxylin–eosin staining, × 400. (**b**) terminal deoxynucleotidyl transferase nick end labelling, × 400. Apoptotic bodies were marked with arrows. Data performed as median (25th percentile, 75th percentile), n = 8 for each group. *P < 0.05 compared with the NC group, ^#^P < 0.05 compared with the M1 group.

The cell layer and number of CA1 and CA3 regions decreased considerably in the M3 group. Decreased cell layer and number of CA1 regions were also observed in the M2 group. The CA3 region cell gaps considerably increased, and the partial cells became smaller with concentrated cytoplasm and nuclei stained deeply in the M2 group. The cell gaps of the CA1 region slightly increased, and the CA1 region cell layer and number of partial cells also slightly decreased in the M1 group (Fig. 2a).

An apoptotic cell is defined as a pyramidal cell with prominent yellowish-brown granules or plaques in the nucleus. The apoptotic bodies were marked with arrows. In the CA1 regions, the apoptosis index of the M3 group was higher than those of M1 (*P* = 0.043) and NC (*P* = 0.009) groups. We could not find the statistical difference in the apoptosis index in the CA3 regions (H = 1.732, *P* = 0.630). Hence, we concluded that the CA1 region of the M3 group had more extensive pathological damage than those of the NC and M1 groups (Fig. 2b).

### Oxidative stress

**Figure 3:**
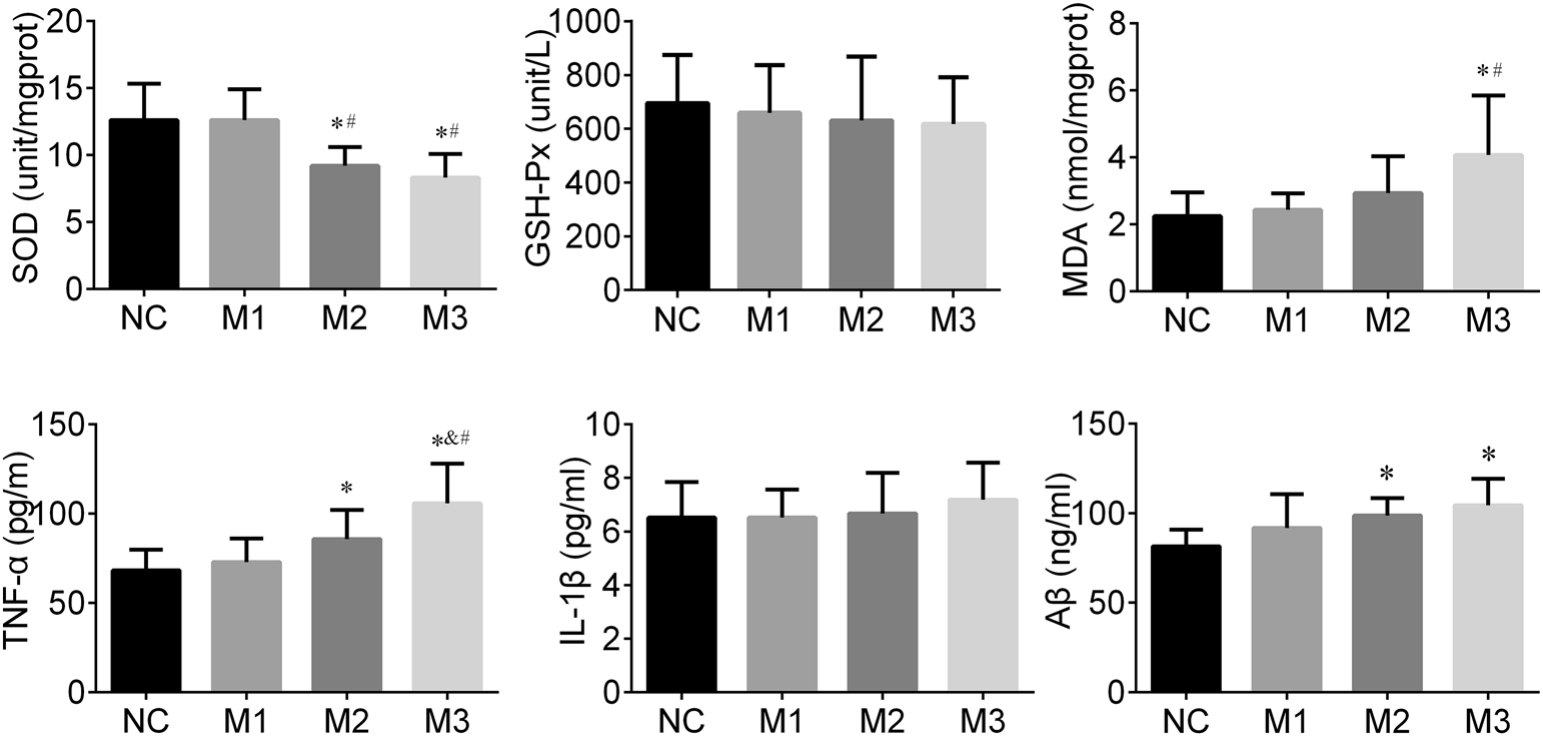
The hippocampal oxidative stress, inflammation cytokines and Aβ of rats. Data is calculated as mean ± standard deviation, n = 8 for each group.

SOD, GSH-Px and MDA are common indicators of oxidative stress. In this study, the different SOD (F = 9.045, *P* < 0.001) and MDA (F = 4.197, *P* = 0.014) levels of the hippocampus were observed among groups. The SOD levels in the M3 group were higher than those in the M1 (*P* < 0.001) and NC group (*P* < 0.001). SOD concentrations in the M2 group were higher than those in the M1 (*P* = 0.003) and NC group (*P* = 0.003). The MDA levels of the M3 group were greater than those of the M1 (*P* = 0.007) and NC group (*P* = 0.003). We did not find significant differences in GSH-Px levels among the groups (F = 0.254, *P* = 0.858). In general, rats in the M3 and M2 groups had higher of oxidative stress levels than those in the NC and M1 groups (Fig. 3).

### Inflammation cytokines

IL-1β and TNF-α are known as classic inflammatory cytokines. In this study, the hippocampal TNF-α level in the M3 group was higher than those in M2 (*P* = 0.021), M1 (*P* < 0.001), and NC (*P* < 0.001) groups. The TNF-α level of the hippocampus in the M2 (*P* = 0.042) group was also greater than that in the NC group. We did not find a significant difference in IL-1β levels among groups (F = 0.454, *P* = 0.716). In general, the hippocampal tissues of the M3 and M2 groups had a stronger inflammation response than that of the NC and M1 group (Fig. 3).

### Aβ

Aβ has been reported to induce Alzheimer’s-like learning and memory impairments. In this study, the hippocampal Aβ level among groups showed differences (F = 4.141, *P* = 0.015), the Aβ levels in the M3 (*P* = 0.002) and M2 (*P* = 0.018) groups were higher than that of the NC group (Fig. 3).

### Associations between SUA at week 48, oxidative stress, inflammation cytokines and Aβ

**Figure 4:**
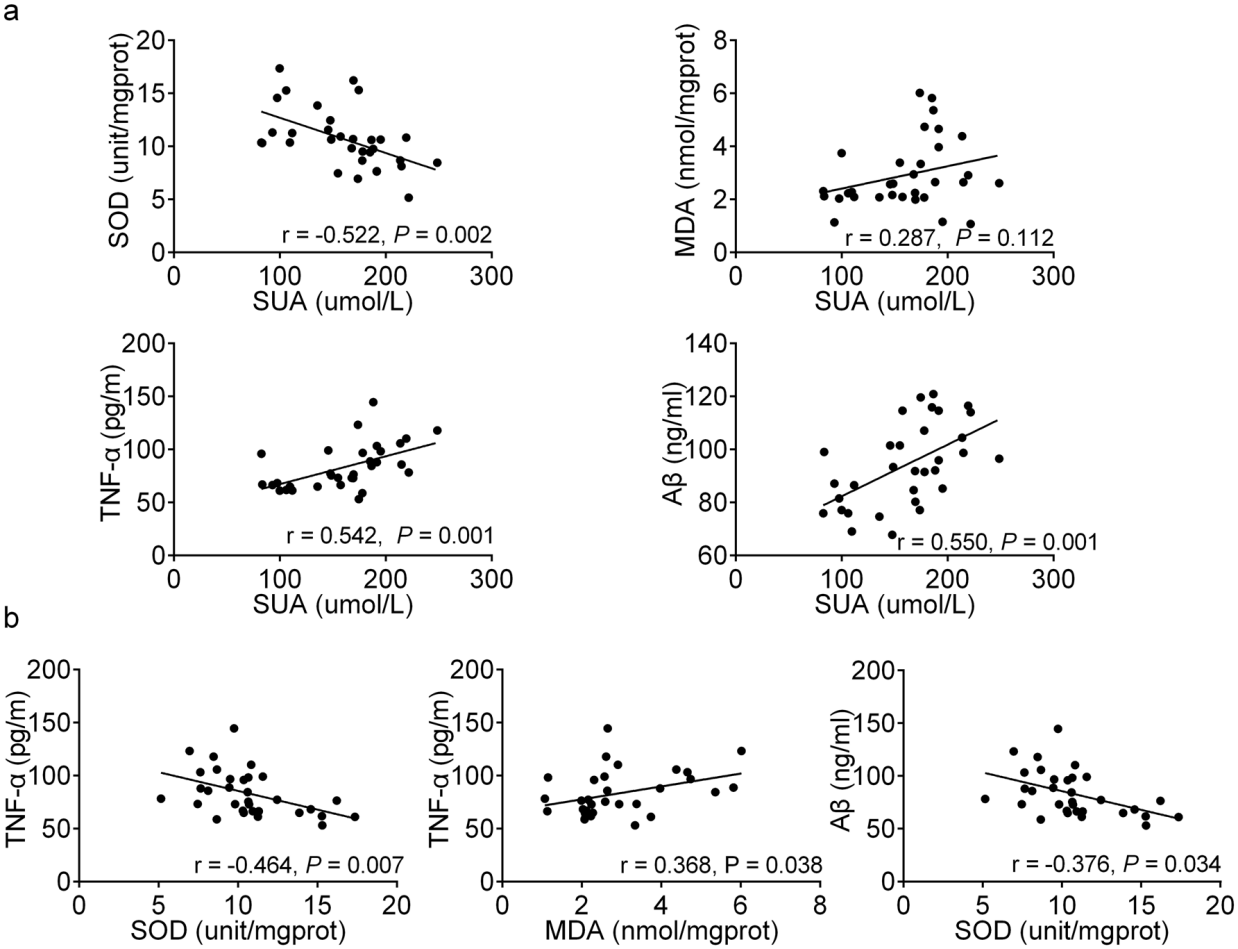
Associations between SUA at week 48, oxidative stress, inflammation cytokines and Aβ. n (subjects’ count) = 32.

Pearson’s correlation analysis showed that there was a significantly negative correlation between SUA and SOD (r = − 0.552, *P* = 0.002), a significantly positive correlation between SUA and TNF-α (r = 0.542, *P* = 0.001), a significantly positive correlation between SUA and Aβ (r = 0.550, *P* = 0.001) (Fig. 4a). Moreover, we also found that TNF-α was associated significantly with SOD (r = − 0.464, *P* = 0.007) and MDA (r = 0.368, *P* = 0.038), and Aβ was significantly correlated with SOD (r = -0.376, *P* = 0.034) (Fig. 4b). We found that the higher the SUA level, the stronger was the response of oxidative stress and inflammation, and the higher the Aβ level. The stronger response to oxidative stress, the stronger the inflammatory response and the higher the Aβ level.

## Discussion

The association between cognitive impairment, a chronic neurodegenerative disorder, and uric acid has been extensively studied, but the results are conflicting. Our data provided clear evidence for the detrimental impact of long-term elevated SUA on cognitive function. Long-term elevated SUA may induce oxidative stress and increase the expression of TNF-α and Aβ in the rat hippocampus.

MWM test is one of the most used methods for evaluating the rat’s spatial learning and memory abilities. The hippocampal CA1 and CA3 regions are the critical brain regions for learning and memory, and damage in this area may contribute to cognitive impairment^23^. In our study, the rats in M3 and M2 groups had longer escape latencies and mean distances to the platform than those of the NC group, indicating poor learning ability under long-term elevated SUA. The histopathology results also showed that the CA1 region of the M3 group had more damage than that of the NC group. These findings suggested the detrimental effect of long-term elevated SUA on cognition to a certain extent and were consistent with several previous studies. Vannorsdall et al. reported that high SUA concentration was related to cognitive impairment in healthy community-dwelling older women^24^. In a prospective cohort study, higher baseline SUA was associated with faster cognitive decline over time^17^. Also, elevated SUA was correlated with greater matter atrophy and cerebral ischemia, which might mediate the association between SUA and cognitive function^16,25^.

Cerebral tissue is susceptible to oxidative damage due to its high oxygen requirement for metabolism, high level of polyunsaturated fatty acids, and low concentration of antioxidant resource^4,26^. The exact mechanisms of how uric acid affects cognition remain unknown; however, oxidative stress is one of the critical factors contributing to the occurrence and development of neurodegenerative disorders^27^. The uric acid concentration in the cerebrospinal fluid depends mainly on the SUA concentration and an impaired blood–brain barrier^28^. Uric acid in serum could pass through the blood–brain barrier and be deposited at a significant level in the hippocampus^19^. Cerebral tissue also could produce uric acid under certain conditions of brain ischemia^29^. Uric acid stimulates the production of hydrogen peroxide^30^. The enzymatic degradation of xanthine also produces superoxide anions during the generation process of uric acid^24^. The imbalance between reactive oxygen species production and elimination could induce oxidative stress, which will eventually cause cell damage and apoptosis^31^. SOD and MAD are the commonly sensitive indexes of oxidative stress in a clinical setting^32,33^. In this study, we observed lower SOD levels and higher MDA levels of hippocampal regions in the M3 and M2 groups than those of the NC group. SUA level was significantly negatively correlated with SOD level, indicating the increased oxidative stress induced by uric acid. Our results were consistent with a previous study’s findings, which detected the hippocampal SOD activity in hyperuricemia rats and found uric acid decreased SOD activity by 19%^19^. Shimamura et al. demonstrated that oxidative stress correlated negatively with cognitive function and positively with postoperative ischemic lesions in carotid artery stenosis stenting^34^.

Hippocampal inflammation is considered to be a risk factor for neurodegenerative disorders^35^. It could trigger reactive gliosis involving the microglia and astroglia^19^. Reactive microglia and astroglia can release inflammation cytokines and chemokines, thereby enhancing the inflammatory response of the brain^36^. Long-term hippocampal modifications induced by inflammation have a crucial impact on brain excitability, associated with neurological dysfunction^37^. The inflammation responses also cause neuronal death and blockade of neurogenesis, thus leading to cognitive impairment^38,39^. In this study, we observed higher hippocampal TNF-α levels in M3 and M2 groups under sustained elevated SUA than those in the NC group, indicating the occurrence of hippocampal inflammation. This finding was consistent with previous studies. Shao et al. study showed that a high uric acid diet increased hippocampal inflammation and reactive gliosis in rodents^19^. Uric acid may induce hippocampal inflammation by activating TLR4/NF-κB signalling, enhancing the inflammatory cytokine gene expression and the upregulation of TNF-α and IL-1β levels^19^.

We also found that TNF-α level was significantly correlated with the hippocampal oxidative stress response in rats. Many research results suggest that the oxidative stress induced by uric acid is like a second messenger for mediators of inflammation^40^. Elevated uric acid can induce reactive oxygen species in vascular endothelial cells, and massive accumulation of reactive oxygen species can upregulate the expression of TNF-α and IL-6^41^. A recent study regarding the treatment of severe gouty arthritis with allopurinol and febuxostat found that MDA and nicotinamide adenine dinucleotide phosphate oxidase levels decreased when uric acid was reduced, suggesting oxidative stress may be involved in the inflammation induced by uric acid^42^.

Aβ consists of 39–42 amino acid residues and is generated from a sequent cleavage of amyloid precursor protein by β-secretase and γ-secretase^43^. Aβ is the major component of an amyloid plaque, one of the hallmarks of Alzheimer’s disease. Our study found Aβ level was negatively correlated with the SOD level and positively correlated with the SUA level. Zlokovic first proposed the idea that age-related vascular injury preceded neurodegeneration and cognitive impairment^44^. Uric acid affects the structure and function of the brain vascular system through oxidative stress and increases the risk of cerebral ischemia^45^. Cerebral ischemia causes endothelial cells to stimulate AβPP/β-secretase expression, leading to an increased Aβ production^46^. Meanwhile, impaired endothelial cells induce the abnormal transmembrane Aβ transport proteins expression, causing the abnormal Aβ transport proteins to cross the blood–brain barrier, resulting in over-deposition of hematogenous Aβ in the brain^47^. The soluble Aβ oligomers are the major cause of synaptic dysfunction and ultimately neurodegeneration^48^. Aβ plaques can also activate astrocytes and microglia. Activated astrocytes and microglia migrate and surround the plaque, release inflammation-associated proteins in the brain, thus causing neuroinflammation^49,50^. In addition, Aβ promotes oxidative stress and apoptosis, which leads to cognitive impairment. Elevated SUA levels may amplify these effects of Aβ^51^.

In this study, no significant differences in escape latencies, mean distances to the platform, levels of Aβ, oxidative stress and inflammation cytokines were observed between the M1 and NC groups, although the M1 group always had a higher SUA level. The pathological injuries in the hippocampal CA1 and CA3 regions of M1 group were also slight compared to the NC group. These results may indicate that the detrimental effect of elevated SUA on cognitive function was probably expressed when the SUA concentration reaches a certain level.

Our results in terms of histopathology, oxidative stress, TNF-α and Aβ in rat hippocampus provided relatively direct evidence for the detrimental effect of elevated SUA on cognitive function. However, several limitations should be noted. First, as we did not observe the histopathology, oxidative stress, inflammation cytokines and Aβ in the intermediate process due to the small sample size restriction, we could not identify the SUA concentration at which pathological changes of the hippocampus occurred. Second, we did not observe a statistical difference in frequencies among the groups, although rats with higher SUA had lower frequencies. However, the damage of the hippocampal CA1 and CA3 regions were evident in the M3 and M2 groups. A significant difference in frequencies might be registered if the sample size would be increased. Third, this study did not involve research of the signalling pathways between oxidative stress and TNF-α and Aβ in the hippocampus. Further research is needed to confirm our findings.

## Conclusion

Long-term elevated serum uric acid was significantly associated with cognitive impairment risk. Oxidative stress, tumour necrosis factor-α and β-amyloid peptide may mediate the pathogenesis of the cognitive impairment induced by uric acid. The detrimental effect of elevated serum uric acid on cognitive function was probably expressed when the serum uric acid concentration reached a certain level.

## Data Availability

The data used to support the findings of this study are available from the corresponding authors upon request.

## Abbreviations

SUA: Serum uric acid
MWM: Morris water maze
SOD: Superoxide dismutase
GSH-Px: Glutathione peroxidase
MDA: Malondialdehyde
IL-1β: Interleukin-1β
TNF-α: Tumor necrosis factor-α
Aβ: β-Amyloid peptide
